# High-dose hypofractionated X-ray radiotherapy for hepatocellular carcinoma: Tumor responses and toxicities

**DOI:** 10.3892/ol.2013.1582

**Published:** 2013-09-12

**Authors:** BING-SHEN HUANG, NGAN-MING TSANG, SHI-MING LIN, DENG-YN LIN, JAU-MIN LIEN, CHEN-CHUN LIN, WEI-TING CHEN, WAN-YU CHEN, JI-HONG HONG

**Affiliations:** 1 Department of Radiation Oncology, Chang Gung Memorial Hospital and Chang Gung University, Taoyuan, Taiwan; 2 Graduate Institute of Clinical Medicine, Chang Gung University, Taoyuan, Taiwan; 3 Department of Gastroenterology and Hepatology, Chang Gung Memorial Hospital and Chang Gung University, Taoyuan, Taiwan; 4 Division of Radiation Oncology, Department of Oncology, National Taiwan University Hospital, Taipei, Taiwan; 5 Graduate Institute of Clinical Medicine, College of Medicine, National Taiwan University, Taipei, Taiwan

**Keywords:** hepatocellular carcinoma, hypofractionated, radiotherapy, toxicities

## Abstract

Hypofractionated radiotherapy (RT) has been employed to treat hepatocellular carcinoma (HCC). The present study aimed to report the treatment effects, the dose-response associations and the factors that are associated with radiation-induced liver disease (RILD) in a high-dose hypofractionated RT procedure. A total of 40 patients with non-metastatic HCC who underwent RT for local control of irradiated tumors were studied. The treatment technique was that of three-dimensional conformal or intensity-modulated radiation therapy, with a fraction size of 3 Gy and a total dose of 40–66 Gy in 14–23 fractions. The biologically-effective dose (BED) was 52.0–85.8 Gy_10_ (median, 74.1 Gy_10_). Tumor regression was observed in 28 patients (70.0%) with a complete response, partial response, stable disease and progressive disease status in 11 (27.5%), 17 (42.5%), five (12.5%) and seven patients (17.5%), respectively. The one-, two- and five-year overall survival (OS) and in-field control (IFC) rates were 60, 40 and 21% and 73, 62 and 56%, respectively. A positive correlation also emerged between the radiation dose and the IFC (P=0.035). Eight of the 40 patients (20%) developed non-classic RILD. A higher Cancer of the Liver Italian Program score was associated with a higher probability of non-classic RILD (P=0.02). The tumor response and IFC rate of HCC following irradiation were significantly dose-dependent. High-dose hypofractionated X-ray RT is a feasible and effective treatment for HCC in patients with good liver function and for those who meet the criteria for a curative attempt.

## Introduction

Hepatocellular carcinoma (HCC) is one of the most common cancers in Asia, where chronic viral hepatitis is common (1). Patients with HCC typically have impaired liver function due to virus- or alcohol-induced cirrhosis or viral hepatitis and only ~20% are appropriate candidates for surgery (2). The five-year overall survival (OS) rate for patients that are treated by surgery is 30–70% (3). For those who are not treated with surgery, liver function affected by an underlying liver disease has a strong affect on the clinical outcomes and complicates treatment strategies to a greater extent than for other tumors. Maximal preservation of the normal liver volume and function is a significant consideration in the choice of treatment.

Percutaneous ethanol injection therapy (PEIT) and radiofrequency ablation (RFA) are two major non-surgical local treatments for HCC. PEIT is often used for small HCCs. Higher local failure rates have been identified in patients with tumors of >3 cm that have been treated by PEIT, or in those with more than three tumors (4). RFA, which is able to treat tumors of ≤5 cm, has a more efficient local control rate than PEIT for small tumors (5). However, RFA is difficult to perform in patients with anatomically unfavorable tumor locations or coagulopathy, as is commonly observed in HCC patients. Transcatheter arterial chemoembolization (TACE), although not considered a curative treatment, is used in patients with poor liver function or those who are not suitable candidates for RFA or PEIT. A systemic review of randomized trials has shown that TACE improves the survival of patients with unresectable HCC (6).

Radiotherapy (RT) has not been widely adopted as a curative treatment modality for HCC due to poor liver tolerance from radiation damage. Improvements in RT techniques, including three-dimensional conformal RT (3DCRT), intensity-modulated RT (IMRT) and image-guided RT (IGRT), provide multiple treatment portals with a reduced volume of liver subjected to high-dose therapy and improved conformity and precision. These techniques increase the prescribed dose and local-control likelihood with acceptable liver toxicity (7). The parallel arrangement of liver-tissue functional subunits has facilitated the employment of hypofractionated RT. A large fraction of HCC was used in proton beam therapy as the normal liver dose may be reduced by its physical characteristic (8). For X-ray, a large fraction size for primary or metastatic liver tumors is provided through stereotactic body RT (9), and clinical trials are being conducted (10).

The present study investigated the use of X-rays with a moderate hypofractionation schema to achieve local control of the irradiated tumor in the treatment of HCC patients. The schema was 3 Gy/fraction, with a maximal total dose of up to 60–66 Gy if the liver tolerance was acceptable. The total dose of this schema was between the conventional fraction size of 2 Gy and the large fraction size provided by stereotactic body radiosurgery.

## Materials and methods

### Patients

The study procedure conformed to the ethical guidelines of the Declaration of Helsinki and approval for the study was obtained from the institution’s human research committee (nos. 99–1,924B). Between January 1998 and January 2008, medical records were reviewed for 40 patients with non-metastatic HCC who underwent high-dose RT, with attention to local tumor control. All the patients were treated with 3DCRT or IMRT and were administered a total radiation dose of >50 Gy_10_. The patients who were diagnosed with HCC and administered RT of a biologically-effective dose (BED) of >50 Gy_10_ using the α/β ratio of 10 Gy were selected for the present study.

Table I provides a summary of the characteristics of the 40 patients, consisting of 10 males and 30 females, with a median age of 63 years (range, 42–82 years). A total of 32 (80.0%) patients presented with liver cirrhosis (LC) and 15 (37.5%) had a history of esophageal variceal (EV) bleeding. Of the 40 patients, 25 (62.5%) had Child-Pugh class A (11) LC and 23 patients (57.5%) had an Eastern Cooperative Oncology Group (ECOG) performance status of 0–1. The previous treatments of the 28 patients who were administered RT as a salvage treatment are as follows: Surgery in one patient, TACE in 23, RFA in two, PEIT in 11 and oral chemotherapy in one. The distribution of patients with a Cancer of the Liver Italian program (CLIP) (12) score of 0, 1, 2, 3 and 4 was six, 13, 13, six and two patients, respectively. Portal vein thrombosis (PVT) was present in 13 patients (32.5%).

### Radiation therapy

The patients were immobilized in a supine position using a vacuum bag with their arms elevated overhead. Contrast-enhanced images were used for target delineation and dynamic computed tomography (CT) was performed as required for tumor identification. The CT images that were captured subsequent to 2007 were obtained by respiration-gating or 4D techniques. The delineation of the gross tumor volume (GTV) accounted for the organ motion in the 4D CT. The clinical tumor volume (CTV) was obtained by adding a 5–10-mm expansion from the GTV, and the expansion of the planning tumor volume (PTV) was typically 5 mm for the lateral directions, 0.5–1 cm for the anterior-posterior direction and 0.5–1.5 cm for the cephalic-caudal direction. The PTV extension depended on 4D or respiratory-gating CT and whether imaged-guided or respiratory gating was used in the treatment. Of the 40 patients, 15 (37.5%), 7 (17.5%) and 18 (45.0%) patients underwent 3DCRT, IMRT and 4D planning RT, respectively.

The prescribed dose was defined as a 100% and 95%, which applied to the CTV and PTV, respectively. The total dose was adjusted by considering the liver tolerance dose with the restriction that <30% of normal liver received >30 Gy (V30) and the dose restriction was reduced to 27 Gy (V27) for those with Child-Pugh class B disease. The median fraction size was 3 Gy/fraction and the radiation dose was 40–66 Gy in 14–23 fractions (BED of 52.0–85.8 Gy_10_ using the α/β ratio of 10 Gy; median, 74.1 Gy_10_). The fraction size was reduced to 2–2.5 Gy if the bowel was included in the PTV. The median of the mean liver dose for all the patients was 2,062 cGy (range, 1,008–2,415 cGy). The median of V30 for all the patients was 24% (range, 12–35%).

### Follow-up

The patient cases were followed-up at least every three months by CT or ultrasonography during the first year and every six months for up to three years thereafter. The follow-up imaging studies were compared with those that were taken prior to RT and the most significant change in tumor size was regarded as the treatment response. The radiographical tumor response following RT was evaluated using the World Health Organization criteria (13). In-field failure (IFF) was defined as tumor regrowth within the current RT field. Intrahepatic recurrence outside the RT field was defined as an intrahepatic failure (IHF). Distant metastasis (DM) was defined as any recurrence outside the liver.

Classic radiation-induced liver disease (RILD) was defined by the presence of anicteric ascites and the elevation of alkaline phosphatase levels to at least a two-fold increase over the pre-treatment values in the absence of tumor progression. The end-point (occurrence of classic RILD) occurred in patients with good liver function. Non-classic RILD was defined as the elevation of alkaline phosphatase levels to more than five times the upper limit of normal or a decline in liver function (measured by a worsening of the Child-Pugh score by two or more). The end-point was described in patients with poor liver function (virus hepatitis, liver cirrhosis, portal hypertension and Child-Pugh Classes B and C) (14).

### Statistics

A univariate cox regression analysis was performed to evaluate the prognostic factors and a multivariate analysis was performed with the forward stepwise procedure using a multiple Cox regression analysis. Survival and IFC were estimated from the first date of RT and the OS rates, and IFC rates were estimated using the Kaplan-Meier method. The Cox regression model was used to investigate the correlation between BED and the IFC. Fisher’s exact test and the logistic regression model were also used to evaluate the correlation between the presence of non-classic RILD and the CLIP score. P<0.05 was considered to indicate a statistically significant difference.

## Results

### Failure pattern and survival

**Figure 1:**
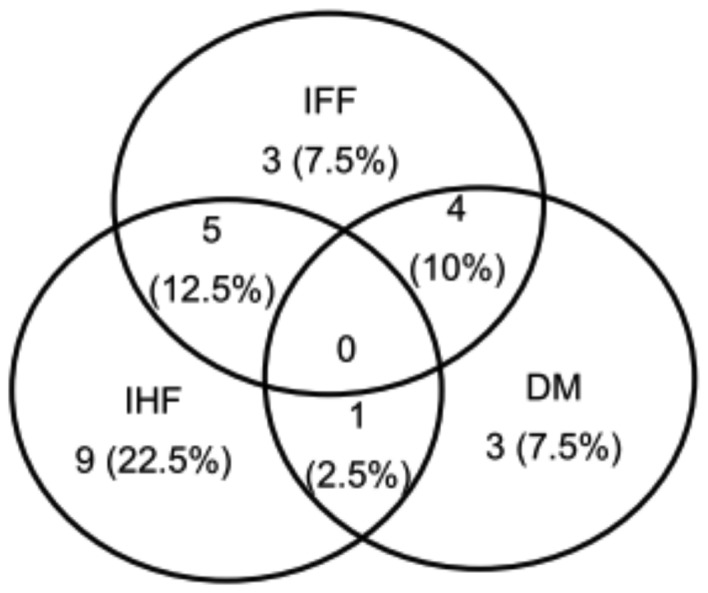
Pattern of failure. The numbers depict cumulative failure sites. IFF, in-field failure; IHF, intrahepatic failure; DM, distant metastasis.

**Figure 2:**
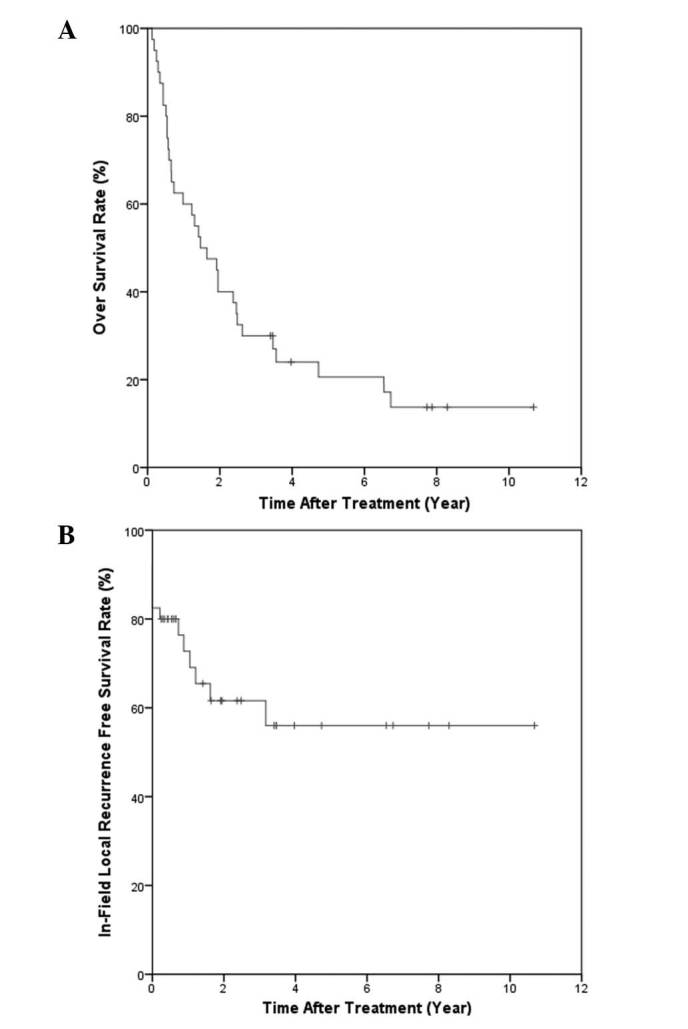
Kaplan-Meier curves of (A) survival and (B) local control. The 1-, 2- and 5-year OS rates were 60, 40 and 21%, respectively. The 1-, 2- and 5-year local control rates were 72.7, 61.6 and 56.0%, respectively.

The failure pattern following a minimum of a two-year follow-up period is shown in Fig. 1. IFF, IHF and DM were observed in 12, 15 and eight patients, respectively, and 10 patients experienced more than one type of recurrence. Following a median follow-up time of 7.7 years for the surviving patients, the one-, two- and five-year OS rates were 60, 40 and 21%, respectively (Fig. 2A). OS was significantly affected by the ECOG performance status (P=0.012), the Child-Pugh classification (P=0.003), the presence of LC (P=0.020), the CLIP score (P=0.001) and the tumor number (P=0.021) in the univariate analysis (Table II). The multivariate analysis showed that Child-Pugh classification (Child-Pugh class B vs. A: HR, 5.42; 95% CI, 2.27–12.95; P<0.0001) and the tumor number (multiple vs. single: HR, 4.68; 95% CI, 2.08–10.53; P<0.0001) were the most significant factors affecting OS. The one-, two- and five-year IFC were 72.7, 61.6 and 56.0%, respectively (Fig. 2B). As shown by the univariate analysis, the factors that were associated with IFC included the tumor number (P=0.026), treatment response and BED (≥60 Gy_10_ vs. <60 Gy_10_, P=0.021; ≥55 Gy_10_ vs. <55 Gy_10_; P=0.001) (Table II). The multivariate analysis revealed that the treatment response (responder vs. non-responder: HR, 0.27; 95% CI, 0.09–0.83; P=0.023) and BED (≥55 Gy_10_ vs. <55 Gy_10_: HR, 0.16; 95% CI, 0.05–0.55; P=0.023) were the most significant factors for IFC. The one-, two- and five-year intrahepatic control (IHC) and distant-metastasis free survival (DMFS) were 65.4, 56.3 and 41.7% and 79.8, 75.3 and 75.3%, respectively. No factors associated with IHC were identified. The treatment response alone affected the DMFS (P=0.032) in the univariate analysis.

### Tumor response

Of the 40 patients, 11 (27.5%) achieved a complete response (CR) following RT and a partial response (PR) was noted in 17 (42.5%) patients. The overall response rate was 70.0%. Stable disease (SD) was observed in five patients (12.5%) and progressive disease (PD) in seven patients (17.5%) (Table III). A positive correlation trend existed between the radiation dose and the tumor response. A higher BED indicated a higher probability of IFC. Using the Cox regression model, the estimated two-year IFC rates for a BED of <60 Gy_10_, 60–70 Gy_10_ and >70 Gy_10_ were 43, 55 and 70%, respectively (P=0.035).

### Toxicity

Eight of the 40 patients (20%) were noted to experience a deterioration of the Child-Pugh score by two or more. The median time of non-classic RILD occurrence from RT completion was 39.5 days (range, 15–85 days). Among the patients who developed non-classic RILD, one (12.5%), five (62.5%) and two (25%) demonstrated CR, PR and PD, respectively. Six of the eight patients with non-classic RILD exhibited ascites and an increased serum total bilirubin level. Using the logistic regression model, the estimated probability of non-classic RILD for CLIP scores 0, 1, 2, 3 and 4 were 3, 8.2, 21, 43.9 and 69.8%, respectively (P=0.02). A higher CLIP score was associated with a higher probability of non-classic RILD. A positive association between BED to the tumor and non-classic RILD was not identified by the dose constraints. The probability of non-classic RILD did not increase when the BED to the tumor increased. However, the mean liver dose for the patients who developed RILD was significantly higher than that for the non-RILD patients (2,322 cGy vs. 1764 cGy; P=0.048). Classic RILD was not noted in any patients. One patient had a duodenal ulcer confirmed by panendoscopy. The patient who developed the duodenal ulcer underwent 3DCRT with a dose of 54 Gy in 18 fractions to PTV and the ulcer location was in the 90–95% isodose region.

## Discussion

RT by X-ray is not routinely used in the curative treatment of HCC. However, HCC has been observed to be more radiosensitive than was previously believed (15). The major limitation has been the poor radiation tolerance of the adjacent normal liver. The first study of the correlation between the dose and complication rate for whole-liver RT was reported by Ingold *et al*, who demonstrated that the RILD incidence was 12.5 and 44% for patients who were treated with 30–35 Gy and >35 Gy whole liver RT, respectively (16). In a Radiation Therapy Oncology Group study for liver metastasis, no RILD was observed in patients who were administered 30 Gy whole liver irradiation provided by 1.5 Gy/per fraction in two factions per day (17). Lawrence *et al*(18) revealed that a higher radiation dose (30 Gy whole liver irradiation with a 15 or 30 Gy boost) resulted in a higher tumor response than whole liver RT alone (64% for the boost group and 39% for whole liver RT alone). In addition, it has been shown that the tolerance for liver irradiation may be 35 Gy for the whole liver, 42 Gy for 70% of the liver, 52 Gy for 50% of the liver and 70 Gy for 30% of the liver (19).

Dawson *et al*(20) showed that liver doses associated with a 5% risk of RILD for uniform irradiation of one-third, two-thirds and the whole liver were 90, 47 and 31 Gy, respectively. Advancements in RT technology have created the possibility of delivering a higher local radiation dose to the liver tumor. A positive correlation between radiation dose and tumor response was observed by Park *et al*(21). The response rates for doses of <40, 40–50 and >50 Gy were 29, 69 and 77%, respectively. The dose response was established in <50 Gy radiation. A PR was observed in 90% of patients with tumors of <5 cm, but only in 60% of those with tumors >5 cm. Park *et al*(7) showed that the IFF rate was 46.7 vs. 16.9% for patients treated with doses of ≤50 Gy_10_ and >50 Gy_10_, and concluded that a BED of 50 Gy_10_ was a criterion for an effective radiation dose. Liu *et al*(22) showed that an improved OS rate was correlated with the dose delivered to the tumor, particularly for doses of >50.4 Gy (1.8 Gy per fraction). Although previous studies have shown that the radiation dose and tumor size affect the treatment results, only a specific dose obtains an enhanced tumor response.

All 40 patients in the present study were administered a greater radiation dose of 52.0–85.8 Gy_10_ with a median BED of 74.1 Gy_10_, showing a positive correlation between the radiation dose and tumor response. A higher BED indicated a higher probability of a tumor response. In addition, a BED of ≥55 Gy_10_ was significantly associated with an improved IFC rate. The results show this schema is feasible for HCC curative treatment and that a dose-response correlation exists for tumor control.

The corresponding BED for the hypofractionated RT schema was 52.0–85.8 Gy_10_ using the α/β ratio of 10 Gy (median, 74.1 Gy_10_). The doses published from 3DCRT with conventional fractionation for HCC were between 33 and 66 Gy (23). A wide range of response (55–92%), one-year local control (61–78%) and one-year OS (43–61%) rates were reported for these doses. Studies have reported similar results to the present treatment strategy with a fraction size of 3–6 Gy/fx, a total dose of 38–68 Gy (24,25), a 55–70% response rate, a 73–85% one-year local control rate and a 60–100% one-year OS rate. Stereotactic body radiotherapy (SBRT) with a diversified fractionation schema showed a 65–100% one-year local control rate and a 48–93% one-year OS rate (9,26). However, stringent patient-selection criteria for liver function, tumor location, tumor size and the high-technique demand limit the routine use of SBRT. The present data and data from other published studies have shown that high-dose hypofractionated conformal RT is feasible and yields an improved local control compared with 3DCRT conventional fractionation.

Although higher-dose RT for HCC is achievable with careful patient selection and an improved radiation technique, RILD remains a significant complication. Data from Western countries indicate that a mean liver dose of 28 Gy in 2-Gy/fx is associated with a 5% risk of classic RILD, which is characterized by fatigue, weight gain, increased abdominal girth, hepatomegaly, anicteric ascites and an isolated elevation in alkaline phosphatase that is out of proportion with the other liver enzymes (27). For the HCC patients in regions with endemic viral infection, the tolerance dose for RILD was shown to be lower and HBV infection predisposed patients to RILD, particularly for non-classic RILD presenting with jaundice or markedly elevated serum transaminases of more than five times the upper limit of the reference range (28,29). Radiation was shown to induce HBV reactivation, possibly through the bystander effect, and non-classic RILD further complicated the RILD for viral hepatitis-related HCC (30,31).

Eight of the 40 patients in the present study developed non-classic RILD, six of whom had viral hepatitis under the liver-dose constraints of V30 <30%. The most significant prognostic factor for non-classic RILD was a high CLIP score. Previous studies have shown that in addition to a normal liver dose and HBV carrier status, the underlying liver function is also a significant predictive factor for RILD (32,33). The Child-Pugh classification has often been used to evaluate liver reserve in cirrhosis patients. The present study revealed that the CLIP score, assigning points for the Child-Pugh score, the tumor morphology (solitary, ≤50% of the liver, massive), the serum α-fetoprotein level and the presence or absence of PVT (12), not only serve as prognostic factors for the survival of HCC patients (34,35), but that they also strongly correlate with RILD incidence.

In the spectrum of local-regional therapy for HCC, the percentage of good surgical candidates for resection is ~20% and the five-year survival rate following surgery is ~50%. RFA results in five-year survival rates of 50%, with up to 20% recurrence rates for larger tumors. However, the result is often limited by the size and location of the tumor. For patients with large or multifocal tumors, TACE offers survival benefits compared with the best supportive treatment alone (36–38). However, the five-year survival rate is <2% and the recurrence rate is nearly 100%. In the present study, ~40% of patients had Child-Pugh class B liver cirrhosis and >75% had viral hepatitis. The tumors in the majority of the patients (70%) failed to respond to previous local-regional therapies and were recurrent multiple HCCs of a moderate size. Within this patient population, the strategy resulted in a five-year IFC rate of 56% and a five-year OS rate of 20.6%. The present study shows that this strategy achieves long-term survival and good local control in certain patients.

In summary, the present study showed that high-dose hypofractionated RT is a feasible and effective treatment for HCC. A positive correlation was identified between the radiation dose and IFC. It was shown that a higher BED indicates a higher probability of IFC. The baseline liver function and the CLIP score should also be evaluated carefully to avoid RILD.

## Figures and Tables

**Table I:** Patient Characteristics.

Characteristic	Number of patients, n (%)
Gender	
Female	10 (25.0)
Male	30 (75.0)
Age, years	
<63	20 (50.0)
≥63	20 (50.0)
ECOG performance status	
0–1	23 (57.5)
2	17 (42.5)
Liver cirrhosis	
No	8 (20.0)
Yes	32 (80.0)
EV bleeding history	
No	25 (62.5)
Yes	15 (37.5)
Child-Pugh class	
A	25 (62.5)
B	15 (37.5)
Previous treatment	
No	12 (30.0)
Yes	28 (70.0)
Surgery	1 (3.6)
TACE	23 (82.1)
RFA	2 (7.1)
PEIT	11 (39.3)
C/T	1 (3.6)
CLIP score	
0	6 (15.0)
1	13 (32.5)
2	13 (32.5)
3	6 (15.0)
4	2 (5.0)
Hepatitis	
NBNC	10 (25.0)
B	6 (15.0)
C	20 (50.0)
B+C	4 (10.0)
Tumor number	
Single	17 (42.5)
Multiple	23 (57.5)
Tumor size, cm	
<5	25 (62.5)
5–10	14 (35.0)
>10	1 (2.5)
PVT	
No	27 (67.5)
Yes	13 (32.5)
AJCC Stage	
I–II	21 (52.5)
III–IV	19 (47.5)

CLIP, Cancer of the Liver Italian Program; C/T, chemotherapy; ECOG, Eastern Cooperative Oncology Group; EV, esophageal varices; NBNC, non-B/non-C; PEI, percutaneous ethanol injection; PVT, portal vein thrombus; RFA, radiofrequency ablation; TACE, transcatheter arterial chemoembolization; AJCC, American Joint Committee on Cancer.

**Table II:** Univariate analysis for OS and IFC.

Clinical feature	2-year OS, %	P-value	2-year IFC, %	P-value
Gender				
Female	10.0		75.0	
Male	50.0	0.151	57.5	0.369
Age, years				
<63	30.0		65.4	
≥63	50.0	0.217	57.1	0.763
ECOG				
0–1	52.2		61.5	
2	23.5	0.012	61.8	0.874
Child-Pugh class				
A	56.0		60.8	
B	13.3	0.003	61.9	0.746
Liver Cirrhosis				
No	62.5		72.9	
Yes	34.4	0.02	58.2	0.763
EV Bleeding				
No	48.0		46.3	
Yes	26.7	0.288	93.3	0.046
CLIP Score				
≥3	12.5		25.0	
<3	46.9	0.019	76.3	0.034
HBV				
No	36.7		57.1	
Yes	50.0	0.227	75.0	0.312
HCV				
No	31.3		39.3	
Yes	45.8	0.522	75.4	0.08
Tumor no.				
Single	58.8		86.9	
Multiple	26.1	0.003	38.6	0.026
Tumor size, cm				
<5	48.0	0.447	74.3	0.402
5–10	28.6	0.479	39.3	0.177
>10	0.0	0.251	-	-
PVT				
No	44.4		66.2	
Yes	30.8	0.286	52.9	0.704
AJCC stage				
I–II	47.6		74.7	
III–IV	31.6	0.062	43.9	0.227
Response				
Non-responder	41.7		31.3	
Responder	39.3	0.624	74.6	0.009

CLIP, Cancer of the Liver Italian Program; ECOG, Eastern Cooperative Oncology Group; EV, esophageal varices; HBV, hepatitis B virus; HCV, hepatitis C virus; IFC, in-field control; OS, overall survival; PVT, portal vein thrombus; AJCC; American Joint Committee on Cancer.

**Table III:** Tumor response to radiation.

Response	Number of patients, (%)
CR	11 (27.5)
PR	17 (42.5)
SD	5 (12.5)
PD	7 (17.5)

CR, complete response; PR, partial response; SD, stable disease; PD, progresive disease.
